# A case report of a child with probable drug resistant tuberculous pericarditis with a review of challenges involved in diagnosis, treatment and follow up of children with DR-TB pericarditis

**DOI:** 10.1186/s12879-020-05027-1

**Published:** 2020-04-22

**Authors:** Aravind Swaminathan, Philipp du Cros, Jay Achar, Jarmila Kliescikova, Shamsiya Mirgayosieva, Bobojon Pirmahmadzoda

**Affiliations:** 1 Médecins Sans Frontières (MSF), Dushanbe, Tajikistan; 2 Clinical Fellow, Paediatric Intensive Care Unit, Great Ormond Street Hospital, London, UK; 3 Médecins Sans Frontières (MSF), Manson Unit, London, UK; 4 National Tuberculosis Programme, Ministry of Health and Social Protection of the Republic of Tajikistan, Dushanbe, Tajikistan

**Keywords:** Case report, Multi drug resistant tuberculosis, Pericarditis; paediatric

## Abstract

**Background:**

There are unique challenges in the diagnosis and management of multi drug resistant tuberculosis (MDR-TB) in children. It is difficult to obtain confirmatory microbiological diagnosis in TB pericarditis. It is essential to differentiate between drug sensitive and drug resistant forms of TB as it has a major bearing on the regimen used, and inappropriate TB treatment combined with steroid use for pericarditis can lead to deterioration. With lack of samples, the treatment decision relies on the drug resistance pattern of the close contact if available. Therapeutic challenges of MDR-TB management in a child involve use of toxic drugs that need to be judiciously handled. We report a 2 years 4 months old male child who was diagnosed with TB pericarditis and treated based on the resistance pattern of his mother who was on treatment for pulmonary MDR-TB.

**Case presentation:**

This 2 years 4 months old male child was diagnosed with TB involving his pericardium. Getting him started on an appropriate regimen was delayed due to the difficulty in establishing microbiological confirmation and drug susceptibility. He was commenced on a regimen based on his mother’s drug resistance pattern and required surgery due to cardiac failure during the course of his treatment. He successfully completed 2 years of therapy.

**Conclusions:**

This child’s case demonstrates that despite unique challenges in diagnosis and management of drug resistant extra pulmonary tuberculosis in children, treatment of even complex forms can be successful. The need for high suspicion of MDR-TB, especially when there is close contact with pulmonary TB, careful design of an effective regimen that is tolerated by the child, indications for invasive surgical management of pericarditis, appropriate follow-up and management of adverse effects are emphasised.

## Background

Globally in 2018, an estimated half million people developed multi-drug or rifampicin resistant tuberculosis (MDR/RR-TB), defined as tuberculosis resistant to rifampicin alone or in combination with isoniazid [1]. Estimates of global annual incidence of MDR/RR-TB in children range between 25,000 and 32,000 [2, 3].

The diagnosis and treatment of childhood MDR/RR-TB suffers from unique challenges particularly amongst younger children. Poor sensitivity and specificity of case finding strategies are compounded by practical difficulties in obtaining diagnostic specimens and inadequate performance of laboratory diagnostic tests. The need for long, toxic treatment regimens alongside limited availability of child-friendly drug formulations are significant barriers to treatment provision [4]. Obstacles to managing MDR/RR-TB in children are exacerbated by the more frequent presence of extra-pulmonary TB (EPTB), up to 25% in children under 4 years [5].

Pericarditis in children could be due to infective (viral, bacterial, mycobacterial) or non-infective causes (autoimmune disease, uraemia, malignancy, drugs or surgery) and presents with substernal chest pain, pericardial friction rub with or without features of cardiac failure [6]. Reports of TB causing pericarditis vary widely from less than 5% of cases in non-endemic settings [7], to about 70% of large pericardial effusions in high prevalence countries [8]. Pericardial TB is more common in combination with immunodeficiency and younger children and is frequently associated with disseminated disease. TB contact history and mycobacterial confirmation are present in the minority of cases [9]. High mortality from TB pericarditis, 17–40%, is likely due to delays in diagnosis, inadequate treatment either prior to drug susceptibility testing (DST) results or where these are not available, and the presence of co-morbid diseases [10]. TB pericarditis is categorized as ‘definite’ when tubercle bacilli are found in stained smear or culture of pericardial fluid and/or tubercle bacilli or caseating granulomata are found on histological examination of pericardium. A diagnosis of ‘probable’ tuberculous pericarditis is made when there is evidence of pericarditis in a patient with tuberculosis demonstrated elsewhere in the body; and/or lymphocytic pericardial exudate with elevated adenosine deaminase (ADA) activity; and/or good response to antituberculosis chemotherapy [11].

Here we present the case of a young child treated successfully for probable DR-TB pericarditis, whose diagnosis and treatment posed many of the above cited challenges. He was diagnosed with “probable tuberculous pericarditis” due to the confirmation of *Mycobacterium tuberculosis* in the pleural fluid accompanied by an otherwise unexplained pericarditis, with a good response to antituberculosis chemotherapy.

## Case presentation

A 2 years 4 months old boy was brought to a TB clinic in Dushanbe, Tajikistan by his mother with prolonged fever, weight loss and decreased general activity. Examination revealed a healthy weight of 13 kg and no clinical features of cardiac failure. His mother was receiving treatment for MDR-TB with additional resistance to second line injectable drugs. There were no other known contacts with TB. Investigations revealed a left-sided pleural effusion with cardiomegaly with no parenchymal abnormalities or lymphadenopathy on chest x-ray and negative human immunodeficiency virus (HIV) test. Three sputum samples were obtained from the child by sputum induction; Ziehl-Nielson microscopy, Xpert® MTB/RIF (Cepheid, Sunnyvale, CA, USA), and mycobacterial cultures were all negative. Echocardiography was unavailable but pericarditis was considered by the clinician. Insufficient volume and the lack of respiratory distress meant the pleural effusion was not aspirated. Following local expert review, in the absence of DST results, he was initiated rifampicin, isoniazid, pyrazinamide and ethambutol for treatment of suspected drug-susceptible TB. Drugs were prescribed according to weight for 8 weeks (three tablets a day of the fixed dose combination comprising rifampicin 75 mg + isoniazid 50 mg + pyrazinamide 150 mg) alongside prednisolone, 2 mg/kg/day, for 4 weeks due to the clinical diagnosis of pericarditis.

Initial resolution of fever and improvement in appetite and activity levels were followed by recurrence of fever and development of respiratory distress, dry cough and generalised oedema suggestive of cardiac failure 6 weeks after starting treatment despite good reported adherence. Echocardiography revealed thickened pericardium (7 mm; a value greater than 4 mm considered to imply constrictive pericarditis [12]) but no effusions or calcifications. Pleural fluid analysis by Genotype MTBDRplus (Hain Lifescience, Nehren, Germany) revealed isoniazid-resistant, rifampicin-sensitive *Mycobacterium tuberculosis*. The same sample was not processed for Mycobacterial culture. In light of this clinical deterioration, his treatment was modified based on the DST of his likely contact and according to local policy. His mother’s phenotypic DST confirmed sensitivity to ethambutol and ofloxacin, and resistance to rifampicin, isoniazid, amikacin, kanamycin and capreomycin.

The decision to start MDR-TB treatment despite a result of rifampicin sensitivity from pleural aspirate was taken due to the clinical deterioration, the mother’s DST, concern about a possible false negative rifampicin resistance test and the potential for amplification of resistance with standard drug sensitive TB treatment given sensitivity to ethambutol and pyrazinamide were not known in the child’s isolate. The boy’s treatment regimen with the corresponding doses are as given in the table below (Table 1):

**Table 1:** TB medications, dose prescribed, dosage per weight and recommended dose range

DRUG	DOSE PRESCRIBED (mg)	DOSE (in mg/kg/day)	DOSE RANGE RECOMMENDED^a^ (in mg/kg/day)
Pyrazinamide	600	46	30–40
Capreomycin	250	19	15–30
Moxifloxacin	100	7.7	7.5–10
Cycloserine	250	19	10–20
Para amino-salicylic acid (PAS)	4000	307	300
Prothionamide	250	19	15–20
Linezolid	250	19	20

^a^Companion Handbook to the WHO guidelines for the programmatic management of drug resistant tuberculosis [13]

Following a 4 weeks course, steroids had already been stopped by the time the boy’s regimen was modified for DR-TB. Since the inflammation in the pericardium seemed well established and not active on echocardiography, steroid was not restarted. Two weeks after completing his steroids, the boy experienced deterioration in cardiac function with increasing oedema and progressive breathlessness. Subtotal pericardiectomy resulted in significant clinical improvement. Biopsies from the excised pericardium were not sent for analysis. Left ventricular ejection fraction improved from 50 to 64% 6 months after surgery alongside improvements in left ventricular end diastolic dimensions. Following discharge after cardiac surgery, his treatment was managed in the community. Since the boy was too young to be cooperative, pure tone audiometry could not be performed. Otoacoustic emission testing was unavailable. In order to provide tailored doses and child-friendly formulations, Médecins Sans Frontières (MSF) supported the Ministry of Health in compounding syrups from quality controlled second line medication (moxifloxacin, prothionamide and pyrazinamide for this child) using a commercially available liquid vehicle.

Clinical response was good and following 2 years of treatment and 1 year of follow-up he was discharged from the program. At discharge, the boy was asymptomatic, achieving appropriate growth mile-stones and had no features of cardiac failure although repeat echocardiography could not be performed.

## Discussion and conclusions

This case highlights a number of obstacles clinicians face when diagnosing and managing a young child suspected of suffering with DR-TB pericarditis.

Pericardial involvement by tuberculosis occurs by lymphatic, hematogenous or contiguous spread. The bacillus penetrating the pericardium provokes a predominantly Th 1 hypersensitive immune response. The ensuing pathological changes in the affected pericardium passes through four stages- fibrinous exudation with an initial polymorphonuclear leucocytosis, serosanguineous effusion with a predominantly lymphocytic exudates, absorption of effusion with organization of granulomatous caseation and pericardial thickening and finally culminating in constrictive thickening of pericardium leading to impairment in diastolic filling of heart [11]. Confirming a diagnosis of TB pericarditis in children is challenging due to practical difficulties in diagnostic sampling, its paucibacillary nature, and limited access to and the non-specific findings of echocardiography. The Tygerberg diagnostic score based on clinical features (fever, night sweats and weight loss) and laboratory parameters (serum globulin level and leucocyte count) has been described with a score greater than 5 indicating a high likelihood of TB pericarditis [14]. When diagnostic sampling is possible, the performance of Xpert® MTB/RIF has shown promise. A recent Cochrane review found that the pooled sensitivity and specificity of Xpert® MTB/RIF on pericardial fluid were 66 and 96% respectively [15]. When compared to microscopy, where sensitivity is reported between 0 and 42%, and culture which may take weeks to provide results, Xpert® MTB/RIF is an important clinical decision-making tool. Unfortunately, in the described case, specimens from surgery were not sent to the laboratory for analysis precluding diagnostic confirmation.

Management of TB pericarditis requires a multi-faceted approach with surgery, corticosteroids and antibiotic therapy all considered beneficial. Surgical drainage and pericardiectomy can been used where constriction or tamponade are detected. Open surgical drainage may be associated with less life-threatening re-accumulation requiring repeat pericardiocentesis in HIV negative patients [16]. Early pericardiectomy has been recommended to be of benefit to avert spread of inflammation to the myocardium and development of fibrosis and calcification of the pericardium [17]. Mitral regurgitation can worsen after pericardiectomy secondary to an increase in the mobility of the left ventricular lateral wall [18]. In this case, deteriorating cardiac function with pericardial thickening prompted surgical subtotal pericardiectomy resulting in marked clinical improvement.

Despite low certainty in the evidence, adjuvant corticosteroids are conditionally recommended by the World Health Organisation (WHO) for people being treated for tuberculous pericarditis [19]. A recent systematic review found some evidence that corticosteroids reduced mortality amongst HIV-negative patients but had little association with constriction [16]. Much of the available evidence includes high proportions of people living with HIV who were untreated with antiretroviral therapy making conclusions difficult to generalise. Use of corticosteroids during empiric treatment of DS-TB may result in worsening of undiagnosed MDR/RR-TB [20, 21].

Lack of DST results from children suffering from possible DR-TB requires clinicians to consider the contact history and the risk of transmission where contacts are multiple. Despite clinical guidelines recommending consideration of the source case’s DST results [22], initial treatment for DS-TB is often started in the absence of detection of drug resistance resulting in additional morbidity and mortality. Since laboratory confirmation of TB is only made in 20–40% of children with radiological evidence of intrathoracic TB [23], in regions where the prevalence of drug-resistance is high a significant proportion of children will start inappropriate treatment when using this approach. In the described case, clinical deterioration 6 weeks after the start of DS-TB treatment could be attributed to inadequate initial treatment. In light of the severity of disease and the young age of the child, the boy was started on treatment for MDR/RR-TB despite DST discordance between pleural fluid and the mother’s isolate.

Although it is generally recommended to use the source case’s DST result to guide empirical therapy in children who lack their own DST result, the use of second-line TB drugs is associated with high risk of toxicity. Overall 30% of patients require cessation of a drug during treatment for MDR/RR-TB due to adverse events [24]. The frequency of ototoxicity in patients treated with injectables for MDR/RR-TB is reported as 10–50% with higher detection noted when monitoring was more stringent [25]. Linezolid is reported to cause reversible myelosuppression amongst 6.4% of children even when duration of therapy is short [26]. Amongst patients receiving linezolid for MDR/RR-TB 55% suffered with adverse events and 35% required dose adjustment or cessation due to severe toxicity [27]. Several instances of peripheral and optic neuropathy have been reported in children, some of which have improved following drug cessation [28]. In addition to toxicity, lack of availability of child-friendly formulations for key second-line TB drugs has been a major barrier to regimen administration [29]. Recently, quality assured formulations have become available for levofloxacin, moxifloxacin, ethionamide, pyrazinamide and cycloserine [30].

A treatment regimen selected using updated global recommendations [31] containing bedaquiline, or delamanid for children younger than 6 years, linezolid, a later generation fluoroquinolone, clofazimine and cycloserine is likely to be more effective in children treated for EP MDR/RR-TB. However, limited experience using these drugs in children under 5 years, a paucity of data on their activity in EPTB, cardiac adverse effects (which might be important in an already diseased heart), lack of paediatric drug friendly formulations and insufficient dosing recommendations in smaller children are also factors to be considered.

This boy’s diagnosis, treatment and follow-up had unique challenges that were overcome with multi-disciplinary teamwork, an individualized patient centred approach and comprehensive care. Barriers to confirming the diagnosis of EPTB and detecting drug resistance in children complicates treatment regimen choices. Attention to the DST of close contacts is essential when selecting an effective treatment regimen.

The reporting of this child is therefore important to highlight the significance of diagnosing and initiating appropriate care promptly for children with MDR/RR-TB pericarditis.

## Data Availability

All data are contained in the manuscript.

## Abbreviations

DR-TB: Drug resistant tuberculosis
MDR-TB: Multi Drug resistant tuberculosis
MDR/RR-TB: Multi Drug resistant/ Rifampicin Resistant tuberculosis
EPTB: Extra Pulmonary tuberculosis
EP MDR/RR-TB: Extrapulmonary Multi Drug resistant/ Rifampicin Resistant tuberculosis
ECHO: Echocardiography
MSF: Medecins Sans Frontieres
MoH: Ministry of Health
R: Rifampicin
H: Isoniazid
Z: Pyrazinamide
E: Ethambutol
Ofx: Ofloxacin
Mfx: Moxifloxacin
Km: Kanamycin
Am: Amikacin
Cm: Capreomycin
S: Streptomycin
PAS: Para amino salicylic acid
